# NIN—at the heart of NItrogen-fixing Nodule symbiosis

**DOI:** 10.3389/fpls.2023.1284720

**Published:** 2024-01-12

**Authors:** Lisha Shen, Jian Feng

**Affiliations:** ^1^ Key Laboratory of Seed Innovation, Institute of Genetics and Developmental Biology, Chinese Academy of Sciences, Beijing, China; ^2^ College of Advanced Agricultural Sciences, University of Chinese Academy of Sciences, Beijing, China; ^3^ CAS−JIC Centre of Excellence for Plant and Microbial Science (CEPAMS), Institute of Genetics and Developmental Biology, Chinese Academy of Sciences, Beijing, China

**Keywords:** nitrogen-fixing symbiosis, *NODULE INCEPTION*, infection thread, nodule organogenesis, autoregulation of nodulation

## Abstract

Legumes and actinorhizal plants establish symbiotic relationships with nitrogen-fixing bacteria, resulting in the formation of nodules. Nodules create an ideal environment for nitrogenase to convert atmospheric nitrogen into biological available ammonia. NODULE INCEPTION (NIN) is an indispensable transcription factor for all aspects of nodule symbiosis. Moreover, NIN is consistently lost in non-nodulating species over evolutions. Here we focus on recent advances in the signaling mechanisms of NIN during nodulation and discuss the role of NIN in the evolution of nitrogen-fixing nodule symbiosis.

## Introduction

Nitrogen is an indispensable nutrient for plant growth. The earth’s atmosphere contains approximately 78% nitrogen. However, atmospheric nitrogen (N_2_) cannot be directly utilized by most plants. Only some plant species from Fabales, Fagales, Cucurbitales, and Rosales (FaFaCuRo) clades exhibit the ability to establish symbiotic interactions with soil nitrogen-fixing bacteria, which are referred to rhizobia or *Frankia* (Soltis et al., 1995; Kistner and Parniske, 2002). This mutualistic symbiosis occurs within specialized structures known as nodules. Rhizobia in nodules utilize the catalytic activity of nitrogenase to convert atmospheric dinitrogen into ammonium, which serves as a nitrogen source for the host plant. In return, the host plant reciprocates by providing rhizobia with carbon sources (Santi et al., 2013; Stambulska and Bayliak, 2019). The establishment of symbiotic interactions requires communications and signal processing between host plants and bacterial partners. These molecular dialogues have been extensively studied in legumes, with a particular focus on model plants such as *Medicago truncatula* and *Lotus japonicus* (Roy et al., 2020; Wang et al., 2022).

Initially, legumes produce and release flavonoid compounds into the rhizosphere in nitrogen-deficient soils, serving as a signal to attract and stimulate rhizobia to produce oligosaccharide, known as Nod factors, thereby initiating a dialogue with the host plant (Zipfel and Oldroyd, 2017). Subsequently, Nod factors are recognized by specific types of receptor kinases, including LjNFR1 (NOD FACTOR RECEPTOR)/MtLYK3 (LysM RECEPTOR KINASE 3), LjNFR5/MtNFP (NOD FACTOR PERCEPTION), and LjSYMRK (SYMBIOTIC RECEPTOR LIKE KINASE)/MtDMI2 (DOSE NOT MAKE INFECTION), which form homomeric and heteromeric complexes on the plasma membrane of root hair cells, triggering the nitrogen-fixing symbiotic signaling pathway (Endre et al., 2002; Stracke et al., 2002; Limpens et al., 2003; Madsen et al., 2003; Radutoiu et al., 2003; Arrighi et al., 2006; Smit et al., 2007; Broghammer et al., 2012; Moling et al., 2014). The perception of Nod factors by receptor kinases located on the membrane transmits the signal to the cell interior, leading to periodic fluctuations in the calcium concentration, referred as calcium spiking, in the nuclei of epidermal root hair cells, which depends on the collaboration of nuclear membranes-localized calcium channel proteins LjPOLLUX/MtDMI1, LjCASTOR, MtCNGC15 (CYCLIC NUCLEOTIDE GATED CHANNEL) and MCA8 (*M. truncatula* calcium ATPase) (Ané et al., 2004; Imaizumi-Anraku et al., 2005; Charpentier et al., 2008; Capoen et al., 2011; Charpentier et al., 2016). The occurrence of nuclear calcium spiking serves as a hallmark event for the activation of the symbiotic signaling pathway (Oldroyd and Downie, 2004). Following that, a calcium and calmodulin-dependent serine/threonine protein kinase, LjCCaMK/MtDMI3, is activated upon to decode the calcium signals, resulting in the phosphorylation of transcription factor LjCYCLOPS/MtIPD3 (INTERACTING PROTEIN OF DMI3) (Lévy et al., 2004; Yano et al., 2008; Singh and Parniske, 2012; Miller et al., 2013; Yuan et al., 2022). Activated LjCYCLOPS/MtIPD3 forms a transcriptional complex with NSP1, NSP2 (NODULATION SIGNALING PATHWAY) and DELLA proteins. Then, this complex promotes the expression of key transcription factor *NIN*, thus initiating NIN-regulated transcriptional network (Singh et al., 2014; Jin et al., 2016). Notably, NIN is one of the earliest-activated transcription factors downstream of common symbiotic signaling pathway (Schiessl et al., 2019).

**Figure 1:**
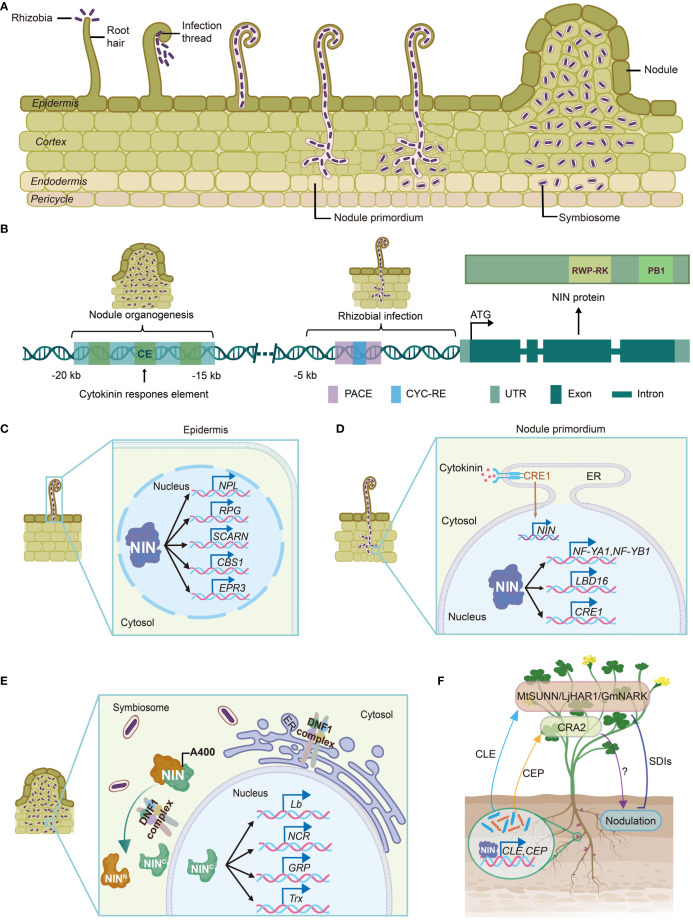
The transcription factor NIN plays essential roles in nitrogen-fixing nodulation. **(A)** Rhizobial infection and nodule organogenesis. Rhizobia enter the host plant through root hairs, which trap the bacteria inside. Afterwards, infection threads are formed and permit invasion of the rhizobia into inner root tissues. Nodule meristem initiates below the infection site in the cortex. The epidermal and cortical processes are coordinated to allow successful intracellular accommodation of rhizobia. Then infection threads then release membrane-bound rhizobia cells into nodule, where the bacteria differentiate and transit into nitrogen-fixing state. **(B)** *NIN* promoter harbors several cis-elements crucial for both rhizobial infection and nodule organogenesis. The CYC-RE and PACE elements in *LjNIN* promoter are required for infection thread development. The green boxes indicate three conserved regions identified in *MtNIN* promoter. The CE region in *MtNIN* promoter is essential for nodule organogenesis. **(C)** NIN facilitates rhizobial infection by regulating expression of infection-associated genes, such as *NPL*, *RPG*, *SCARN*, *CBS1* and *EPR3*. *NPL* encodes a pectate lyase enzyme involved in cell wall restructuring during rhizobia invasion. RPG is a critical determinant for the formation of infectosome, which is a protein complex essential for infection thread development. *SCARN* encodes a nodulation-specific component of the SCAR/WAVE complex. CBS1 contains a cystathionine-β-synthase (CBS) domain and a domain of unknown function. EPR3 is a LysM receptor that recognizes exopolysaccharides on the surface of rhizobia, promoting infection thread initiation. **(D)** NIN is essential for nodule organogenesis. NIN drives the expression of *NF-YA1* and *NF-YB1*, inducing cortical cell division and nodule primordium formation. NIN also controls the expression of *LBD16*, a key transcription factor involved in lateral root development, which has been hijacked to coordinate nodule development. Additionally, activated cytokinin receptor CRE1 promotes *NIN* expression in nodule primordium. Then, cortical NIN proteins activate *CRE1* expression, forming a positive feedback loop. **(E)** NIN determines the cellular state transition to nitrogen fixation. The DNF1-complex mediates the processing of NIN protein at A400, generating a C-terminal NIN fragment, which specifically activates a suit of genes involved in symbiosome development and nitrogen fixation, such as *Lb*, *NCR*, *GRP*, and *Trx*. **(F)** NIN controls AON signaling. NIN activates the expression of CLE and CEP peptides in root, which are subsequently transported to shoot. After perceived by MtSUNN/LjHAR1/GmNARK receptors, the CLE peptides activate the production of SDIs, which move back to root and suppress further nodulation. CEP peptides are recognized by shoot receptor CRA2, resulting in promotion of nodulation. Created with medpeer.cn. NIN, NODULE INCEPTION; CYC-RE, CYCLOPS-responsive element; PACE, Predisposition-Associated Cis-regulatory Element; CE, cytokinin response element-containing region; NPL, NODULATION PECTATE LYASE; RPG, RHIZOBIUM-DIRECTED POLAR GROWTH; EPR3, EXOPOLYSACCHARIDE RECEPTOR 3; ER, endoplasmic reticulum; NF-Y, NUCLEAR FACTOR-Y SUBUNIT; LBD16, LATERAL ORGAN BOUNDARIES DOMAIN 16; CRE1, CYTOKININ RESPONSE 1; DNF1, Defective in Nitrogen Fixation 1; Lb, leghemoglobin; NCR, nodule specific cysteine-rich; GRP, glycine-rich peptide; Trx, thioredoxin; AON, autoregulation of nodulation; CLE, CLAVATA3/EMBRYO SURROUNDING REGION; CEP, C-terminally Encoded Peptide; SUNN, SUPERNUMERARY NODULES; HAR1, HYPERNODULATION AND ABERRANT ROOT; NARK, NODULE AUTOREGULATION RECEPTOR KINASE; SDI, shoot-derived inhibitor; CRA2, COMPACT ROOT ARCHITECTURE2.

The transcription factor NIN belongs to a plant-specific RWP-RK protein family. NIN controls all aspects of symbiotic nodulation in legumes: rhizobial infection, nodule organogenesis, transition to nitrogen fixation, and regulation of nodule number in legumes and actinorhizal plants (**Figure 1**) (Schauser et al., 1999; Borisov et al., 2003; Marsh et al., 2007; Soyano et al., 2013; Soyano et al., 2014; Clavijo et al., 2015; Liu C.W. et al., 2019; Liu J. et al., 2019b; Bu et al., 2020; Feng et al., 2021). Moreover, *NIN* gene is consistently lost or unfunctional in some non-nodulating species of FaFaCuRo clades, suggesting that the nitrogen-fixing ability of plants may associate with functional NIN protein (Griesmann et al., 2018; van Velzen et al., 2018; Zhang et al., 2023). In this review, we focus on recent advances in understanding the regulatory mechanisms of NIN in nodule symbiosis and discuss the evolution of *NIN* function in nitrogen-fixing nodule (NFN) symbiosis.

## The transcription factor NIN: structure and function

The transcription factor NIN was initially identified in *L. japonicus* by forward genetic screening (Schauser et al., 1999). *nin* mutations block the rhizobial entry at an early stage (**Table 1**) (Schauser et al., 1999; Borisov et al., 2003; Marsh et al., 2007; Feng et al., 2021). CYCLOPS activates *NIN* expression by binding to the CYCLOPS-responsive elements (CYC-RE or PACE) in the *NIN* promoter (Singh et al., 2014; Cathebras et al., 2022). The CYC-RE in *NIN* promoter is conserved in legumes (Liu C. W. et al., 2019). It was recently reported that a putative CYC-RE was also present in the promoter of one poplar *NIN* ortholog (Irving et al., 2022), suggesting that this element may be recruited before the origin of nodulation. Both CYC-RE and PACE are critical for *NIN* function during infection thread development (**Figure 1B**) (Liu J. et al., 2019; Akamatsu et al., 2022; Cathebras et al., 2022). Additionally, another cis-element CE (cytokinin response element-containing region) was reported to be essential for nodule organogenesis and *NIN* expression in the pericycle (**Figure 1B**) (Liu J. et al., 2019). The CE is not present outside nodulated legumes (Liu and Bisseling, 2020; Zhang et al., 2023), suggesting that original recruitment of *NIN* into nodulation may occur in CYC-RE, whereas CE-dependent induction of *NIN* in inner root cell layers may evolve later.

**Table 1:** List of published *nin* alleles in nodulating plants.

Species	Allele	Mutant line	Background	Phenotypic defects			Mutagen	Genomic mutation	Effect of mutation	Reference
				Infection	Nodule organogenesis	Nitrogen fixation				
*Lotus japonicus*	*Ljnin-1*	96.1M2	Gifu	n/a	Nod-	n/a	*Ac* transposon insertion	Transposon insertion at C1459	*Ac* transposon insertion (unstable)	Schauser et al., 1999
	*Ljnin-2*	96.1M2 offspring 1	Gifu	n/a	Nod-	n/a	*Ac* transposon insertion	Transposon insertion at C1459	Frame shift	Schauser et al., 1999
	*Ljnin-3*	96.1M2 offspring 2	Gifu	n/a	Nod-	n/a	*Ac* transposon insertion	Transposon insertion at C1459	Frame shift	Schauser et al., 1999
	*Ljnin-4*	96.1M2 offspring 3	Gifu	n/a	Nod+	n/a	*Ac* transposon insertion	Transposon insertion at C1459	Amino acid insertion (V410-N411)	Schauser et al., 1999
	*Ljnin-5*	96.1M2 offspring 4	Gifu	n/a	Nod+	n/a	*Ac* transposon insertion	Transposon insertion at C1459	Amino acid insertion (N410)	Schauser et al., 1999
	*Ljnin-6*	KL773	Gifu	n/a	Nod+	n/a	*Ac* transposon insertion	Transposon insertion at C1459	Amino acid insertion (V410-N411)	Perry et al., 2009
	*Ljnin-7*	*sym47*	Gifu	Inf-	n/a	n/a	Lotus retrotransposon 1 insertion	Transposon insertion at G2599	n/a	Madsen et al., 2005
		KL577	n/a	n/a	Nod-	n/a	n/a	n/a	n/a	Sandal et al., 2006
	*Ljnin-8*	B21-1^a^; B47-B	Gifu	n/a	Inf-	n/a	EMS	C1785 to T	Q519 to stop condon	Murray et al., 2006; Perry et al., 2009
	*Ljnin-9*	SL5369^b^; SL5426^c^	Gifu	n/a	Nod+; Nod-	n/a	EMS	G1002 to A	V258 to M	Perry et al., 2009
		n/a	MG-20	n/a	Nod-	n/a	EMS	A nucleotide substitution from G2017 to A at splice donor site	n/a	Suzaki et al., 2012
	*Ljnin-10*	S46-1^d^	Gifu	n/a	Nod-	n/a	EMS	G1242 to A	A338 to T	Murray et al., 2006; Perry et al., 2009
	*Ljnin-11*	SL0605-2, 3^e^	Gifu	n/a	Nod-	n/a	EMS	G1848 to A	E540 to K	Perry et al., 2009
	*Ljnin-12*	SL1798-2, 4, 5	Gifu	n/a	Nod+	n/a	EMS	G1927 to A	R566 to K	Perry et al., 2009
	*Ljnin-13*	SL3012-1	Gifu	n/a	n/a	n/a	EMS	G2431 to A	G685 to R	Perry et al., 2009
	*Ljnin-14*	SL5800-3	Gifu	n/a	Nod-	n/a	EMS	G986 to A	E252 to E	Perry et al., 2009
	*Ljnin-15*	n/a	Gifu	Inf-	Nod+	n/a	Lotus retrotransposon 1 insertion	Transposon insertion at *NIN* promoter 143 bp of 3’ PACE element	n/a	Cathebras et al., 2022
	*daphne*	n/a	MG-20	Inf+	Nod-	n/a	Carbon ion beam irradiation	A reciprocal chromosomal translocation at approximately 7 kb upstream of the start codon of *NIN*	Knock down	Yoro et al., 2014
*Medicago truncatula*	*Mtnin-1*	12S	A17	Inf-	Nod-	n/a	Fast neutron bombardment	An 11 bp deletion at 1850 bp	Frame shift leads to premature termination	Marsh et al., 2007
	*Mtnin-2*	Tnt148	R108	Inf-	Nod-	n/a	*Tnt1* transposon insertion	Transposon insertion at 20 bp upstream of the start codon of *NIN*	Knock down	Marsh et al., 2007
	*Mtnin-3*	Tnk148	R108	n/a	Nod-	n/a	*Tnt1* transposon insertion	Transposon insertion at 26 bp upstream of the start codon of *NIN*	n/a	Pislariu et al., 2012
	*Mtnin-4*	NF2728	R108	n/a	Nod-	n/a	*Tnt1* transposon insertion	Transposon insertion at 617 bp of *NIN*	n/a	Pislariu et al., 2012
	*Mtnin-5*	NF0532	R108	n/a	Nod-	n/a	*Tnt1* transposon insertion	Transposon insertion at 704 bp of *NIN*	n/a	Pislariu et al., 2012
	*Mtnin-6*	NF1317	R108	n/a	Nod-	n/a	*Tnt1* transposon insertion	Transposon insertion at 1196 bp of *NIN*	n/a	Pislariu et al., 2012
	*Mtnin-7*	NF1277	R108	n/a	Nod-	n/a	*Tnt1* transposon insertion	Transposon insertion at 1397 bp of *NIN*	n/a	Pislariu et al., 2012
	*Mtnin-8*	NF1263	R108	n/a	Nod-	n/a	*Tnt1* transposon insertion	Transposon insertion at 1664 bp of *NIN*	n/a	Pislariu et al., 2012
*Medicago truncatula*	*Mtnin-9*	NF3019	R108	n/a	Nod-	n/a	*Tnt1* transposon insertion	Transposon insertion at 1665 bp of *NIN*	n/a	Pislariu et al., 2012
	*Mtnin-10*	NF0117	R108	n/a	Nod-	n/a	*Tnt1* transposon insertion	Transposon insertion at 1977 bp of *NIN*	n/a	Pislariu et al., 2012
	*Mtnin-11*	NF3046	R108	n/a	Nod-	n/a	*Tnt1* transposon insertion	Transposon insertion at 2241 bp of *NIN*	n/a	Pislariu et al., 2012
	*Mtnin-12*	NF2640	R108	n/a	Nod-	n/a	*Tnt1* transposon insertion	Transposon insertion at 2628 bp of *NIN*	n/a	Pislariu et al., 2012
	*Mtnin-13*	NF0440	R108	n/a	Nod+	n/a	*Tnt1* transposon insertion	Transposon insertion at 2647 bp of *NIN*	Frame shift leads to PB1 deletion	Pislariu et al., 2012; Liu et al., 2021
	*Mtnin-14*	NF0825	R108	n/a	Nod-	n/a	*Tnt1* transposon insertion	Transposon insertion at 2819 bp of *NIN*	n/a	Pislariu et al., 2012
	*Mtnin-15*	NF2700	R108	n/a	Nod-	n/a	*Tnt1* transposon insertion	Transposon insertion at 2970 bp of *NIN*	n/a	Pislariu et al., 2012
	*Mtnin-16*	NF10547	R108	n/a	Nod+	n/a	*Tnt1* transposon insertion	Transposon insertion at 2669 bp of *NIN*	Frame shift leads to PB1 deletion	Veerappan et al., 2016; Liu et al., 2021
	*daphne-like*	FN8113	A17	Inf+	Nod-	n/a	Fast neutron bombardment	2.49 Mb chromosome 2 insertion at 4120 bp upstream of the start codon of *NIN*	n/a	Liu J. et al., 2019
*Pisum sativum*	*Psnin/sym35*	SGENod^–^1	SGE	Inf-	Nod-	n/a	EMS	C1657 to T	Q553 to stop condon	Tsyganov et al., 1999; Borisov et al., 2003
	*Psnin/sym35*	SGENod^–^3	SGE	Inf-	Nod-	n/a	EMS	C160 to T	Q54 to stop condon	Tsyganov et al., 1999; Borisov et al., 2003
		RisNod8	Finale	Inf-	Nod-	n/a	EMS	G1210 to A	E404 to K	Engvild, 1987; Borisov et al., 2003
*Glycine max*	*Gmnin1a*	n/a	Williams 82	n/a	Nod-	n/a	RNA interference	n/a	Knock down	Fu et al., 2021
	*Gmnin1b*	n/a	Williams 82	n/a	Nod+	n/a	RNA interference	n/a	Knock down	Fu et al., 2021
	*Gmnin2a*	n/a	Williams 82	n/a	Nod+	n/a	RNA interference	n/a	Knock down	Fu et al., 2021
	*Gmnin2b*	n/a	Williams 82	n/a	Nod+	n/a	RNA interference	n/a	Knock down	Fu et al., 2021
	*Gmnin1b*	n/a	Huachun 6	Inf+	Nod+	n/a	CRISPR-Cas9	C1774 deletion	Frame shift leads to premature termination	Bai et al., 2020; Fu et al., 2022
	*Gmnin1a nin1b*	n/a	Huachun 6	Inf+	Nod+	n/a	CRISPR-Cas9	1 bp insertion at G1773 of *NIN1a*	Frame shift leads to premature termination	Bai et al., 2020; Fu et al., 2022
								1 bp insertion at G1699 of *NIN1b*	Frame shift leads to premature termination	Bai et al., 2020; Fu et al., 2022
	*Gmnin2a nin2b*	n/a	Huachun 6	Inf+	Nod+	n/a	CRISPR-Cas9	83 bp insertion at G1564 of *NIN2a*	Frame shift leads to premature termination	Bai et al., 2020; Fu et al., 2022
								86 bp insertion at G1285 of *NIN2b*	Frame shift leads to premature termination	Bai et al., 2020; Fu et al., 2022
	*Gmnin1a nin2a nin2b*	n/a	Huachun 6	Inf-	Nod-	n/a	CRISPR-Cas9	14 bp deletion at G250 of *NIN1a*	Frame shift leads to premature termination	Bai et al., 2020; Fu et al., 2022
								Insertions at A1540 of *NIN2a*	Frame shift leads to premature termination	Bai et al., 2020; Fu et al., 2022
								G1264 and A1265 deletions and 86 bp insertion at G1285 of *NIN2b*	Frame shift leads to premature termination	Bai et al., 2020; Fu et al., 2022
	*Gmnin1a nin1b nin2a nin2b*	n/a	Huachun 6	Inf-	Nod-	n/a	CRISPR-Cas9	5 bp deletion at G255 of *NIN1a*	Frame shift leads to premature termination	Bai et al., 2020; Fu et al., 2022
								14 bp deletion at G256 of *NIN1b*	Frame shift leads to premature termination	Bai et al., 2020; Fu et al., 2022
								G1543 and A1544 deletions and 83 bp insertion at G1564 of *NIN2b*	Frame shift leads to premature termination	Bai et al., 2020; Fu et al., 2022
								Insertion at A1265 of *NIN2b*	Frame shift leads to premature termination	Bai et al., 2020; Fu et al., 2022
*Cicer arietinum*	*Carn4*	PM405	P502 (ICC 640)	n/a	Nod+	Fix-	γ-ray irradiation	A2189 deletion	Frame shift leads to PB1 deletion	Davis, 1986; Frailey et al., 2022
*Parasponia andersonii*	*Pannin*	B1	WU1.14	n/a	Nod-	n/a	CRISPR-Cas9	Deletions at T181 and A242 of *NIN*	Frame shift leads to stop condon at amino acid position 90	Bu et al., 2020
		B3	WU1.14	n/a	Nod-	n/a	CRISPR-Cas9	Deletion at T181 of *NIN*	Frame shift leads to stop condon at amino acid position 70	Bu et al., 2020

a Line contains in addition a *har1-1* mutation.

b Line SL5369 carries the mutant alleles *nin-9* and *nsp2-9*.

c Line SL5426 carries the mutant alleles *nfr1-6*, *nfr5-6* and *nin-9*.

d Line S46-1 carries the mutant alleles *castor-25*, *nin-10*, and *har1-1*.

e Lines SL0605-2,3 carry the mutant alleles *nin-11* and *symrk-9*.

n/a: not applicable.

NIN protein is characterized by a conserved 60-amino acid-long sequence containing an RWPxRK motif, which exhibits conservation with the MID (minus dominance) protein, the first identified member possessing this motif (Ferris and Goodenough, 1997). The conserved RWPxRK motif was subsequently designated as the RWP-RK domain and categorized as a novel class of transcription factors (Schauser et al., 1999). Structure predictions and a series of protein-DNA binding assays demonstrate the DNA-binding capability of RWP-RK domain, allowing NIN to interact with specific DNA sequences located within the promoters of target genes. These NIN-regulated genes include early nodulation genes such as *NF-YA1* and *NF-YB1* (*NUCLEAR FACTOR-Y SUBUNIT A1*), *NPL* (*NODULATION PECTATE LYASE*), *CRE1* (*CYTOKININ RESPONSE 1*), *ASYMMETRIC LEAVES 2-LIKE 18*/*LATERAL ORGAN BOUNDARIES DOMAIN 16* (*ASL18*/*LBD16*), and late nodulation-associated genes such as leghemoglobins, thioredoxins, nodule-specific cysteine-rich (NCR) peptides and glycine-rich peptides (**Figures 1C–F**) (Xie et al., 2012; Soyano et al., 2013; Vernié et al., 2015; Soyano et al., 2019; Feng et al., 2021).

Another remarkable characteristic of NIN protein is the presence of PB1 (Phox and Bem1) domain at its C-terminal end, which mediates protein-protein interactions, allowing NIN to form dimers or oligomers (**Figure 1B**) (Sumimoto et al., 2007; Feng et al., 2021). NIN-like proteins (NLPs), named after its homology to NIN protein, share the RWP-RK and PB1 domains with NIN, but not its N-terminal nitrate binding domain (Liu et al., 2022). NLPs have been characterized as key regulators of nitrate signaling in land plants (Castaings et al., 2009; Konishi and Yanagisawa, 2013; Marchive et al., 2013; Chardin et al., 2014; Liu et al., 2017; Alvarez et al., 2020; Liu et al., 2022). Interestingly, *M. truncatula* NLP1 and *L. japonicus* NLP1/4 are required for the repression of nodulation by nitrate (Lin et al., 2018; Nishida et al., 2018; Nishida et al., 2021). MtNLP1 interacts with NIN through the PB1 domain, leading to suppression of NIN-activated *CRE1* expression (Lin et al., 2018). Adaptations in *NIN* promoter and functional changes to NIN protein enable its specific functions in NFN symbiosis.

## NIN facilitates intracellular rhizobial infection

The infection thread is crucial for rhizobia invasion into host plant during nodulation. Upon recognition of Nod factors released by rhizobia, root hairs of host plant undergo curling, enclosing the rhizobia attached to the surface of root hairs (Esseling et al., 2003). Subsequently, cell wall surrounding the enclosed rhizobia is locally degraded, and the cytoskeleton in root hair undergoes rearrangement, resulting in the invagination of cell membrane and formation of a tubular structure known as infection thread. Rhizobia gain entry into plant cells through infection threads, extending to the base of the root hair and subsequently penetrating the developing nodule primordia, which arise from differentiated cells composed of cortical cells, endodermis and pericycle (**Figure 1A**) (Oldroyd, 2013).

*NIN* is among the earliest-responding genes to rhizobia inoculation, suggesting its involvement in the initiation of bacterial infection, except rhizobial crack-entry infection in peanut (*Arachis hypogaea*) (Schiessl et al., 2019; Mergaert et al., 2020; Bhattacharjee et al., 2022). *NIN* loss of function leads to widespread defects in gene expression, highlighting its pivotal role in the gene regulatory network governing rhizobia infection (Liu C.W. et al., 2019). Notably, NIN controls many early genes associated with nodulation (**Figure 1C**). Among them, *NPL* encodes a pectate lyase enzyme involved in cell wall restructuring during rhizobia invasion (Xie et al., 2012). RPG (RHIZOBIUM-DIRECTED POLAR GROWTH) is a critical determinant for the formation of an exocyst complex (termed as infectosome) during bacterial infection (Arrighi et al., 2008; Lace et al., 2023). NIN directly binds to the *RPG* promoter and induce its expression (Li et al., 2023). *SCARN* (*SCAR-Nodulation*), a gene responsible for actin rearrangement during rhizobia infection, is induced by rhizobia in epidermal cells and directly regulated by NIN (Qiu et al., 2015). In addition, the absence of the membrane-localized protein CBS1 (cystathionine-β-synthase-like 1) results in the formation of an elevated number of microcolonies, whose expression is dependent on NIN (Sinharoy et al., 2016). NIN also promotes the expression of *EPR3* (*Exopolysaccharide Receptor 3*) in *L. japonicus*, a LysM receptor gene responsible for sensing rhizobia exopolysaccharides and facilitating the entry of rhizobia into host cells (Kawaharada et al., 2015; Kawaharada et al., 2017). These extensive regulations by NIN underscore its central role in orchestrating the early responses to rhizobia invasion in legumes.

## NIN is essential for nodule organogenesis

As root hairs curl and entrap compatible rhizobia, cell divisions in the cortex, endodermis and pericycle are induced, triggering the formation of nodule primordia (Xiao et al., 2014). After successful invasion of legume plants by rhizobia, the initiation of root nodule organogenesis occurs in a coordinated manner to ensure accurate intrusion of infection thread into developing nodule primordium (**Figure 1A**) (Oldroyd and Downie, 2004). Mutants of *NF-YA1* and *NF-YB1*, members of nuclear factor-Y (NF-Y) transcription factor family, show abnormal infection thread development, delayed nodule formation, and smaller nodules, demonstrating their important roles in root nodule development (Laporte et al., 2013; Soyano et al., 2013). Further studies show that NIN directly binds to the promoter regions of *LjNF-YA1* and *LjNF-YB1* genes, promoting their expression (**Figure 1D**). Overexpression of *LjNIN* and *LjNF-YA1* genes induces cell division in the root cortex, resulting in the formation of nodule-like structures (Soyano et al., 2013). *ASL18*/*LBD16* is a key transcription factor that regulates lateral root development by activating expression of auxin synthesis-related genes, thus promoting auxin biosynthesis and influencing lateral root growth (Shahan and Benfey, 2020). Interestingly, the developmental program controlled by ASL18/LBD16 in lateral roots appears to be involved in root nodule organogenesis as well. NIN recruits the core developmental program of lateral roots to facilitate root nodule formation by promoting the expression of *ASL18*/*LBD16* (**Figure 1D**) (Schiessl et al., 2019; Soyano et al., 2019). These findings demonstrate that LBD16 and NF-Y transcription factors act downstream of NIN and work cooperatively to regulate root nodule development (Bishopp and Bennett, 2019).

Phytohormone cytokinin plays key roles in regulating various aspects of plant growth and development. Exogenous application of cytokinin has been shown to induce formation of nodule-like structures in leguminous plants (Gauthier-Coles et al., 2018). In *L. japonicus*, gain-of-function mutants *snf2* and *snf5* (*spontaneous nodule formation*) of the cytokinin receptor gene *LHK1* (*LOTUS HISTIDINE KINASE1*) exhibit a spontaneous nodule phenotype in the absence of rhizobia (Tirichine et al., 2007; Liu et al., 2018). Similarly, overexpression of gain-of-function *CRE1* mutant, a homolog of *LHK1* in *M. truncatula*, also induces rhizobia-free nodule formation (Jin et al., 2016). These results demonstrate the essential role of cytokinin signaling in nodule formation. Interestingly, the *NIN* gene is up-regulated by cytokinin treatment or *snf2* mutation. And spontaneous nodule formation in *snf2* mutants appears a NIN-dependent manner (Tirichine et al., 2007). Consistently, a distal element in *NIN* promoter, containing putative cytokinin B-type response regulator binding sites, is responsible for cytokinin-induced *NIN* expression, which is necessary for nodule organogenesis (**Figure 1B**) (Liu J. et al., 2019b). Furthermore, NIN protein is sufficient to activate the expression of *CRE1*, forming a positive feedback regulatory loop that promotes nodule development (**Figure 1D**) (Vernié et al., 2015). Alongside this, cytokinin and *NIN*-overexpression induced cortical cell divisions are dependent on the GRAS proteins MtSHR (SHORTROOT) and MtSCR (SCARECROW) (Dong et al., 2021). Together, these findings reveal the crucial role of NIN-dependent regulatory network governing root nodule formation in leguminous plants.

## NIN determines the transition to nitrogen fixation

Symbiotic nitrogen fixation requires a low-oxygen environment for proper activity of nitrogenase (Kondorosi et al., 2013). However, the mechanism controlling the transition to nitrogen fixation remains elusive for years. While NIN has been extensively studied for its role in various aspects of nodule initiation and development, recent research reveals that NIN also regulates the transition of nodule cells into nitrogen-fixing state. Nodulation activated signal peptidase complex (SPC) mediates the processing of NIN protein, resulting in production of a C-terminal NIN fragment containing the DNA binding domain. The processed C-terminal product of NIN specifically activates a suite of genes associated with symbiosome development and nitrogen fixation [such as genes encoding leghemoglobins, nodule specific cysteine-rich (NCR) peptides and thioredoxins], thereby controlling the cell state transition (**Figure 1E**) (Feng et al., 2021). In addition, NIN and its close homolog NLP2 directly promote the expression of leghemoglobins, which buffer the oxygen concentration within nodules (Jiang et al., 2021). These findings demonstrate the important roles of NIN and NLP2 in creating suitable environment for nitrogen fixation.

## NIN controls autoregulation of nodulation

Symbiotic nodulation is an energy-consuming process, and excessive nodule formation adversely affects regular development of host plant (Wang et al., 2021; Ke et al., 2022). To maintain energy balance between nitrogen fixation and other developmental processes, nodule number is tightly controlled by autoregulation of nodulation (AON) system. AON signaling pathway consists of root-derived signals, receptors in shoot and shoot-derived inhibitors (SDIs), which involve root-shoot-root communications determining optimal nodule numbers (Roy et al., 2020). Transcription factor NIN activates the expression of *CLE* (*CLAVATA3/EMBRYO SURROUNDING REGION*) peptides to initiate AON (**Figure 1F**) (Soyano et al., 2014; Laffont et al., 2020; Wang et al., 2020). The AON-related CLE peptides are widely present in legumes, including *M. truncatula* CLE12/13, *L. japonicus* CLE-RS1/2 (CLE-ROOT SIGNAL), as well as RIC1/2 (RHIZOBIUM INDUCED CLE) peptides in soybean (*Glycine max*) and common bean (*Phaseolus vulgaris*). As root-derived signals, these CLEs are transported through xylem to shoot (Okamoto et al., 2008; Mortier et al., 2010; Lim et al., 2011), where they are recognized by the leucine-rich-repeat receptor-like kinase (LRR-RLK), termed MtSUNN (SUPER NUMERIC NODULES) in *M. truncatula*, LjHAR1 (HYPER NODULATION ABERRANT ROOT FORMATION 1) in *L. japonicus*, and GmNARK (NODULE AUTOREGULATION RECEPTOR KINASE) in *G. max.* This perception triggers production of SDIs that move back to root suppressing further nodulation (Krusell et al., 2002; Nishimura et al., 2002; Searle et al., 2003; Schnabel et al., 2005; Tsikou et al., 2018). In contrast, *M. truncatula* CEP7 (C-terminally Encoded Peptide), which is induced by rhizobia and cytokinin, plays a crucial role in promoting rhizobia infections and nodule formation through the receptor MtCRA2 (COMPACT ROOT ARCHITECTURE 2) in shoots. Coordinated expression of *CLE* and *CEP* genes by NIN allows precise control of nodule number in plants (**Figure 1F**) (Laffont et al., 2020). In soybean, GmNINa activates the expression of *miR172c*, which relieves the transcriptional repression of *GmRIC1/2* by NNC1 (Nodule Number Control 1), thus activating AON pathway. Conversely, NNC1 represses *miR172c* expression, forming a negative feedback loop. NNC1 also interacts with GmNINa to antagonistically regulate the transcriptional activation of *GmRIC1/2*. Thus, the GmNINa-miR172c-NNC1 signaling axis systemically regulates nodulation and AON signaling (Wang et al., 2019).

## Discussion

Symbiotic associations between plants and nitrogen-fixing microbes shape the global ecosystems during the evolution of life on earth. However, plants forming NFN symbiosis are restricted to the FaFaCuRo families (Griesmann et al., 2018; van Velzen et al., 2018; Zhang et al., 2023). Possibly because NFN symbiosis needs intensive energy to produce nodules and fuel nitrogen-fixing reactions. Alternatively, reduced immune responses allowing rhizobia invasion may make plants susceptible to disease (Mathesius, 2022). Recent phylogenomic studies propose a scenario of single gain of nodulation, followed by multiple losses (van Velzen et al., 2019). The emergence of master regulator NIN from a duplication event of NLP and subsequent evolutionary changes, such as the acquisition of specific promoter elements and/or amino acid substitutions, underscores its adaptive significance in driving the evolution of NFN symbiosis in legumes (Liu J. et al., 2019b; Cathebras et al., 2022; Zhang et al., 2023). More efforts are needed to decipher the molecular changes on NIN protein to enable occurrence of NFN symbiosis. We need to better understand the underlying mechanisms of NIN and NLP in regulating different biological processes that range from NFN symbiosis and nitrate signaling. Knowledge from analysis of fossil samples with root nodules and ancient DNA studies would also provide direct evidence for how the NFN symbiosis origins.

Since this initial discovery, *NIN* has been identified as essential for nodulation in nitrogen-fixing land plants. Besides legumes, NIN orthologs are also required for NFN symbiosis in actinorhizal plants *Parasponia andersonii* and *Casuarina glauca* (Clavijo et al., 2015; Bu et al., 2020). *P. andersonii* hosts rhizobia in thread-like structure, called fixation thread, which is equivalent to symbiosome in legumes that hosts its rhizobial partners. Both structures provide proper environments for nitrogen fixation (Behm et al., 2014). It would be interesting to explore whether the regulatory mechanisms on nodulation and nitrogen fixation mediated by NIN is conserved between legumes and actinorhizal plants. Furthermore, a novel NFN between seagrass *Posidonia oceanica* and N_2_-fixing symbiont has been reported recently (Mohr et al., 2021). This finding makes it possible to test functional conservation of NIN in NFN symbiosis across land and aquatic plants. To obtain a complete picture of mechanisms that control the interactions between plants and nitrogen-fixing microorganisms, more plant-microbe systems are needed to be established. Future investigations of NFN symbiosis occurring across diverse plant species will expand our knowledge of molecular mechanisms for nitrogen-fixing signaling and will allow a strategic initiative towards the transfer of the nitrogen-fixing symbiosis to non-nodulating crops.

## Author contributions

LS: Investigation, Validation, Writing – original draft, Writing – review & editing. JF: Conceptualization, Funding acquisition, Investigation, Project administration, Resources, Supervision, Validation, Visualization, Writing – review & editing.
